# Expression of four phosphate transporter genes from Finger millet (*Eleusine coracana* L.) in response to mycorrhizal colonization and Pi stress

**DOI:** 10.1007/s13205-017-0609-9

**Published:** 2017-04-08

**Authors:** Ramesh Namdeo Pudake, Chandra Mohan Mehta, Tapan Kumar Mohanta, Suvigya Sharma, Ajit Varma, Anil Kumar Sharma

**Affiliations:** 1 Amity Institute of Nanotechnology, Amity University Uttar Pradesh, Sector-125 Campus, Noida, Uttar Pradesh 201 313 India; 2 Department of Biological Sciences, College of Basic Sciences and Humanities, G. B. Pant University of Agriculture and Technology, Pantnagar, Uttarakhand 263 145 India; 3 Amity Institute of Microbial Technology, Amity University Uttar Pradesh, Sector-125 Campus, Noida, Uttar Pradesh 201 313 India; 4 School of Agriculture, Lovely Professional University, Jalandhar-Delhi G.T. Road (NH-1), Phagwara, Punjab 144 402 India; 5 School of Biotechnology, Yeungnam University, Gyeongsan, Gyeongsangbuk-do 38541 Republic of Korea

**Keywords:** Arbuscular mycorrhiza, Phosphate transporter, Pi stress, Gene expression, Symbiosis

## Abstract

Phosphorus (P) is a vital nutrient for plant growth and development, and is absorbed in cells with the help of membrane-spanning inorganic phosphate transporter (Pht) protein. Symbiosis with arbuscular mycorrhiza (AM) also helps in transporting P from the soil to plant and Pht proteins play an important role in it. To understand this phenomenon in Finger Mille plant, we have cloned four *Pht* genes from Finger millet, which shares the homology with Pht1 protein family of cereals. Expression pattern analysis during the AM infection indicated that *EcPT4* gene was AM specific, and its expression was higher in roots where AM colonization percentage was high. The expression level of *EcPT1*-*4* gene under the phosphorous (Pi) stress in seedlings was found to be consistent with its role in acquisition of phosphorus. Homology study of the EcPt proteins with Pht proteins of cereals shows close relationship. The findings of the study indicate that Pht1 family genes from finger millet can serve to be an important resource for the better understanding of phosphorus use efficiency.

## Introduction

Finger millet (*Eleusine coracana* L.) is grown in many parts of the world with a wide range of environmental conditions. Its production ranks sixth in India after wheat, rice, maize, sorghum and pearl millet. It is a good source of mineral nutrients like calcium, phosphorus and also provides amino acids like lysine and methionine for the peoples from Asian and African regions (Dida et al. 2008). The grains are being used for preparation of traditional foods, such as *roti* (bread), *mudde* (dumpling) and *ambali* (thin porridge). It shows antimicrobial, antioxidant and anti-diabetic properties because of the presence of polyphenols in seeds of this millet (Devi et al. 2014). It is the main food grain for many peoples, especially in areas with soil having poor nutrient level (Kumar et al. 2016; Upadhyaya et al. 2007).

Along with other nutrient, phosphorus (Pi) is one of the essential mineral nutrients for proper growth and development of plant. Being a structural component of nucleic acids and phospholipids, it plays an important role in biological processes like photosynthesis, energy transfer reactions, and signal transduction (Li et al. 2010; Versaw and Harrison 2002). The phosphorus is abundantly present in the soil but not in readily available form due to its high fixation rate in the soil. This is a worldwide problem and a limiting factor in agriculture production (Sánchez-Calderón et al. 2010); as 70% of the global cultivated land, including acidic and alkaline calcareous soils, suffers from inorganic phosphate (Pi) deficiency, making Pi nutrition a research area of great priority (Lopez-Arredondo et al. 2014). With the increasing demand for food (http://faostat.fao.org/), the uncontrolled fertilization has given rise to many environmental problems. Hence, developing the eco-friendly technologies for effective use of P under P-limited conditions will be of major importance for agricultural sustainability. The plants acquire phosphorus from the soil solution either directly via absorption by roots or indirectly through a mycorrhizal symbiosis (Richardson 2001; Walder et al. 2015). The past studies have indicated the presence of a mineral transport system in plants that consist of membrane-spanning phosphate transporter family proteins (Pht1 family). The members of this gene family are identified from various plants like *Arabidopsis thaliana* (Bayle et al. 2011; Remy et al. 2012), rice (Ai et al. 2009; Campos-Soriano et al. 2012; Sun et al. 2012; Wang et al. 2014; Wu et al. 2013), wheat (Davies et al. 2002; Duan et al. 2015; Guo et al. 2014), tomato (Chen et al. 2014; Liu et al. 1998a), tobacco (Tan et al. 2012), maize (Nagy et al. 2006; Su et al. 2014), barley (Schünmann et al. 2004), *Medicago truncatula* (Javot et al. 2007; Liu et al. 1998b), *Populus trichocarpa* (Loth-Pereda et al. 2011), and soybean (Inoue et al. 2014; Song et al. 2014).

In our previous study, we have found that some genotypes of finger millet showed differential response in term of growth and yield in the presence of mycorrhizal symbiosis (Unpublished data). This differential response may be due to different genetic factors involved in better establishment of AMF symbiosis. Mainly, the phosphate transporter genes from root have been reported to be involved in nutrient exchange during symbiosis with AMF (Walder et al. 2015). With objective for better understanding of mechanism of phosphate uptake in finger millet, we cloned phosphate transporter genes and studied their expression pattern in association with AMF and in phosphorus stress condition.

## Materials and methods

### Plant material and cultivation conditions

Seeds of finger millet were surface sterilized with 1% sodium hypochloride for 5 min, followed by 70% ethanol for 1 min. The traces of ethanol were removed by repeated washing with sterilized distilled water. Sterile seeds were then germinated on solidified agar without any salt. For colonization with mycorrhiza, 1-week-old uniform seedlings were transplanted to sterile sand: soil mix (4:1) along with 1 g of inoculum of *Glomus intraradices* consisting of spores (50 spores/g), extracellular hyphae, and colonized root fragments. Six seedlings were grown in each pot. The culture of *G. intraradices* was established with maize plant by growing it in vermiculite supplied with Hogland’s solution (Hoagland and Arnon 1950) that is devoid of phosphorus for three cycles of 60 days each. Three varieties of finger millet (Ragi Korchara Local, Khairna, and VHC3611) were grown in greenhouse with and without mycorrhiza. The seedlings were supplemented with Hoagland’s nutrient solution with 1/4th strength of phosphorus. All six seedlings were harvested after 30 days. Half of the plant roots were used for mycorrhiza infection study using trypan blue staining (Phillips and Hayman 1970). The remaining roots and leaves were immediately frozen in liquid nitrogen and stored at −80 °C for RNA extraction.

For the study of Pi stress, 7-day-old seedlings of finger millet (Ragi Korchara Local) were first transferred to sterile vermiculite supplemented with Hoagland’s nutrient solution. After 15 days of growth in vermiculite, the seedlings were transferred to the hydroponic float system in a tray containing 3 l of Hoagland’s nutrient solution with aeroponic pumps. For phosphorus stress, after 1 week the Hoagland’s nutrient solution was replaced with fresh nutrient solution with 1/4th of KH_2_PO_4_. The potassium ions (K) were compensated using K_2_SO_4_ to fulfill the shortage of K. The roots and leaves were harvested in the morning (09.00–10.00 h) after every 2 days (up to 6 days) following the initiation of phosphate starvation. The harvested tissues were immediately frozen in liquid nitrogen and stored at −80 °C until further analysis. All the experiments were repeated with three biological replicates, with six seedlings used for harvesting leaves and roots at a defined time span.

### Detection of AMF colonization

After 30 days of co-culturing, the finger millet plants were harvested and roots were gently washed under running water to remove the adhering potting mixture. After cleaning, roots were immersed in 10% KOH solution and kept in the water bath at 100 °C for 15 min; and later washed with distilled water thrice and dipped in 1 N HCl. The samples were stained with trypan blue, followed by destaining (Phillips and Hayman 1970). The roots were then stored in lactic acid, glycerol, and water (1:1:1 by volume) and checked for AM colonization under a microscope (Olympus BX40, Japan). Magnified line-intersect method (McGonigle et al. 1990) was used for colonization rating.

### RNA isolation and cDNA synthesis

Total RNA was isolated from roots and leaves of finger millet by RNA Express reagent according to the manufacturer’s instructions (Himedia, Mumbai, MS, India). The isolated RNA was treated with RQ1-DNase (Promega, Madison, WI, USA) to ensure that all genomic DNA contamination was removed. First-strand cDNA was synthesized using 2 µg of total RNA with of Oligo (dT_15_) primer (500 ng/μl) and M-MLV reverse transcriptase according to the manufacturer’s protocol (Promega, Madison, WI, USA). The resulting cDNA mixture was diluted to 20 times by adding nuclease-free water and stored at −20 °C until further use. PCR with *Tubulin* gene (CX265249) primers (Gupta et al. 2011) (Table 1) was conducted to ensure that synthesis of cDNA was successful. The amplified PCR fragments were detected by agarose gel electrophoresis. To confirm the complete digestion of genomic DNA by DNAse I, PCRs with *EcTub* gene primers were also performed with non-reverse transcribed total RNA. The failure to amplify the fragment confirmed the removal of genomic DNA from RNA samples.

**Table 1:** Primers used for cloning of full-length EcPT genes and expression study

Code	Sequence	Use
EcPT1-F	5′ CCGCCTCTACTACAGCGAGCCTAACA 3′	Real-Time Q-PCR
EcPT1-R	5′ ACACCACCATGAGAATGAGCGTGAA 3′	
EcPT1-3 N	5′ GTCTGAGTACGCCAACAAGAGAAC 3′	For 3′ RACE PCR
EcPT1-5 N	5′ ACCGCATAGGGCGACACCGTTCA 3′	For 5′ RACE PCR
EcPT2-F	5′ ACACGCCTAAGAGCGTCATTG3′	Real-Time Q-PCR
EcPT2-R	5′ CCACAATTGTGCCGAAGAGGATG3′	
EcPT2-3 N	5′ TCTGAGTACGCCAACAAGAAGAC 3′	For 3′ RACE PCR
EcPT2-5 N	5′ GAGCCAGAACCTGAAGAAGCAGAGC 3′	For 5′ RACE PCR
EcPT3-F	5′ CCGGTCAGCTCTTCTTCGGGTGGCT 3′	Real-Time Q-PCR
EcPT3-R	5′ CGGCGGAGATGATGAGCGTGACAAT 3′	
EcPT3-3 N	5′ ACCTACTACTGGCGGATGAAGAT 3′	For 3′ RACE PCR
EcPT3-5 N	5′CGAGCCAGAAGCGGAAGAAGCAGAG 3′	For 5′ RACE PCR
EcPT4-F	5′ ACGCCTACGACCTATTCTGCATCACC 3′	Real-Time Q-PCR
EcPT4-R	5′ GCCGAACACGAG CTGGCCTATCA 3′	
EcPT4-3	5′ GACATGACCAATGTGATGGAGATC 3′	For 3′ RACE PCR
EcPT4-3 N	5′ CTAGTAGTAACTTCCTTGCTCCAGC 3′	
EcPT4-5 N	5′ CGCCACGCCGATGACCATGTTGT 3′	For 5′ RACE PCR
EcTub-F	5′ CTCCAAGCTTTCTCCCTCCT 3′	Internal Control for expression study
EcTub-R	5′ GCATCATCACCTCCTCCAAT 3′	

### Cloning of phosphate transporters genes from finger millet

For cloning of putative phosphate transporters gene (*EcPT*) from finger millet, all available full-length sequences of rice PT genes were downloaded from GenBank database (Benson et al. 2008). These sequences were compared by Bioedit software (Hall 1999) for sequence similarities. Based on the sequence analysis, required primers were designed and used for amplification of putative fragments of *EcPT* genes. The resultant PCR products were cloned into pGEM-T vector (Promega, Madison, WI, USA) and sequenced. The resulted cDNA sequences were used for searching the homology with phosphate transporter genes using BlastX (McGinnis and Madden 2004). To amplify the full-length cDNAs of partial *EcPT* genes, FirstChoice RLM-RACE Kit (Invitrogen, Grand Island, NY, USA) and gene-specific primers were used (Table 1).

### *In silico* analysis of *EcPT* genes

Multiple alignments of deduced EcPT amino acid sequences were carried out using Multalin software (http://bioinfo.genopole-toulouse.prd.fr/multalin/). Different statistical parameters used during multiple sequence alignment were: sequence input format, fasta; protein weight matrix, Blosum-62-12-2; gap penalty at opening, default; gap penalty at extension, default; gap penalty at extremities, none; one iteration only, no; high consensus value, 90%, and low consensus value, 50%. Transmembrane helices prediction was conducted using the online server TMHMM Server v. 2.0 (Krogh et al. 2001). Molecular modeling of all the phosphate transporter proteins was conducted using online server of SwissModel automatic modeling mode (Schwede et al. 2003). The phylogenetic relationships of deduced amino acid sequences with different phosphate transporter protein family members of rice, maize, and arabidopsis were analyzed using MEGA6 (Larkin et al. 2007; Tamura et al. 2007). Different statistical parameters used to construct the phylogenetic tree were: analysis, phylogeny reconstruction; statistical method, maximum likelihood; no. of bootstrap replicate, 1000; substitution type, amino acid; model/method, Jones-Taylor-Thornton (JTT) model; rates among sites, uniform rates; gaps/missing data treatment, partial deletion; and branch swap filter, very strong.

### qRT-PCR expression analysis of *EcPT* genes

The transcript abundance levels of cloned *EcPT* genes were investigated by quantitative real-time PCR (qPCR) using an MX3005P Real-time PCR system (Stratagene, Santa Clara, CA, USA). The 25.0 μl of reaction mixture contained 12.5 μl Maxima SYBR Green/ROX qPCR master mix (Fermentas, Maryland, USA), 1.0 μl of each primer at 10 μM, 8.5 μl ddH2O, and 2.0 μl (80 ng) cDNA. Full-length gene sequences for *EcPT* genes were used for designing the specific primer pairs (Table 1). Tubulin gene is considered as a housekeeping gene as it is expressed all tissues irrespective of its stages, so it was selected as internal control in qRT-PCR (Gupta et al. 2011). Each sample was amplified in triplicate using an equal amount of cDNA template. The PCR temperature profiles were as follows: an initial step for 10 min at 95 °C; followed by 40 cycles of 30 s at 95 °C, 30 s at 60 °C, and 40 s at 72 °C. Expression levels of the putative phosphate transporter gene (Ct) were calculated using the 2^−ΔΔCT^method (Livak and Schmittgen 2001) using the accompanying software of MX3005P real-time PCR detection system (Stratagene, Santa Clara, CA, USA).

## Results

### Cloning of four phosphate transporter genes (*EcPT1, 2, 3* and *4*)

The rice phosphate transporter genes available in public domain (Jia et al. 2011; Paszkowski et al. 2002) were used to design the Pht1 family gene-specific primers in *Eleusine coracana*. The PCR was carried out with different set of primes (data not shown) that resulted in amplification of four cDNA fragments from *E. coracana* (*EcPT1, 2, 3* and *4*). The sequencing results of these fragment showed identity with phosphate transporter genes of other organism when compared by BlastX (Altschul et al. 1990). The full-length cloning of those four cDNA fragments by Rapid amplification of cDNA ends (RACE) technique was tried to achieve using the adaptor and gene-specific primers. The sequencing results revealed that *EcPT1* was 1886 bp long, including a 5′ and 3′ un-translated region (UTR), and is predicted to contain an open reading frame of 524 amino acids (GenBank accession number KJ842583). The second and third genes, called *EcPT2* and *EcPT3*, were partial with 1274 and 1478 bp in length, respectively, with a predicted 3′ truncated open reading frame of 396 and 470 amino acids (GenBank accession number KJ842584 and KJ842585). The fourth gene *EcPT4* was of 2066 bp in length with a predicted open reading frame of 546 amino acids (GenBank accession number KJ842586).

### *EcPT1*-*4* shares sequence identity with other members of phosphate transporter family

**Fig. 1:**
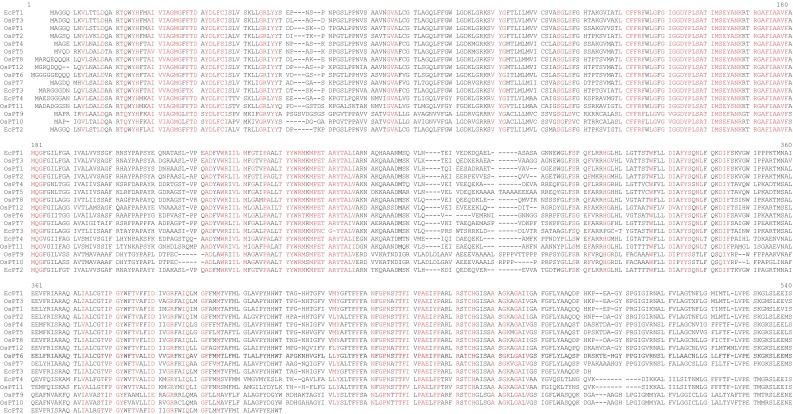
Multiple sequence alignment of EcPT proteins. Multiple sequence alignment shows presence of several conserved domains including A-I-V-I-A-G-M-G-F-x-F-T-D-x–Y-D-L-F-S-I, G-R-x–Y-Y, L-C-F–F-R-F-x-L-G-x-G-I-G–G-D-Y-P-L-S-A-T-I-M-S-E-Y-A-N-K, R-G-A-F-I-A-A-V-F-x-M-Q-G, T-Y-Y-W-R-M-x-M-P-E-T-A-R-Y–T-A-L-I/V, and N-x-G-P-N-x-T–T-F-I-x-P-A-E-x-F-P in EcPT proteins (Underlined by solid line). Besides these conserved domains, EcPT proteins also contain several conserved motifs and amino acids. Multiple sequence alignment of EcPT proteins was conducted using Multalin software (http://multalin.toulouse.inra.fr/multalin/)

**Fig. 2:**
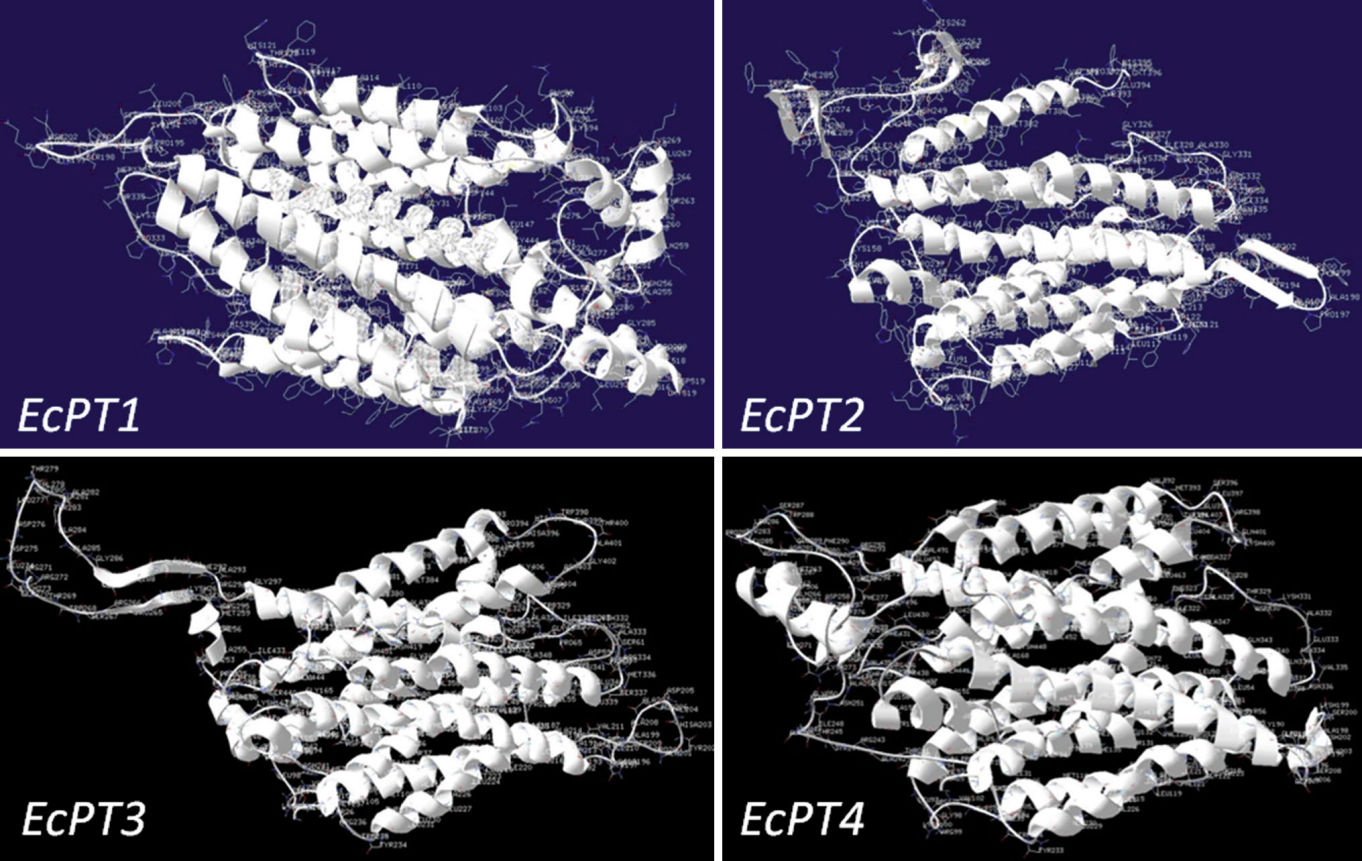
Molecular structures of EcPT proteins. Molecular structure of EcPT proteins was modeled by swiss-model workbench automatic modeling server (http://swissmodel.expasy.org/workspace/index.php?func=modelling_simple1&userid=USERID&token=TOKEN). Deduced protein sequences were used to model the molecular structure. Molecular structure shows the presence of 12 alpha helices in EcPT protein that contains a central cytosolic tunnel that is required to transfer the phosphate molecule. The molecular structure of EcPT protein resembles the molecular structure of eukaryotic phosphate transporter protein

**Fig. 3:**
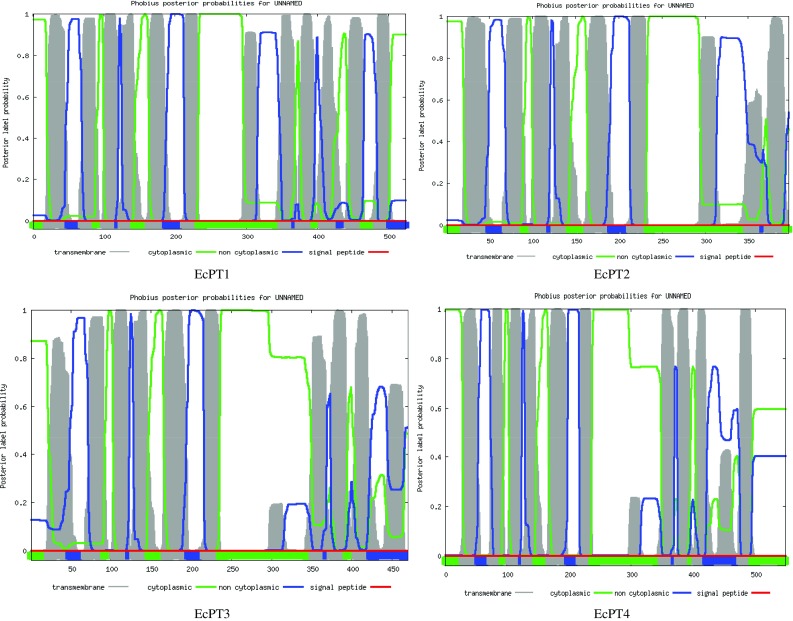
Prediction of transmembrane domain in EcPT using TMHMM server. Result shows the presence of membrane-spanning transmembrane domain in all four EcPT proteins

**Fig. 4:**
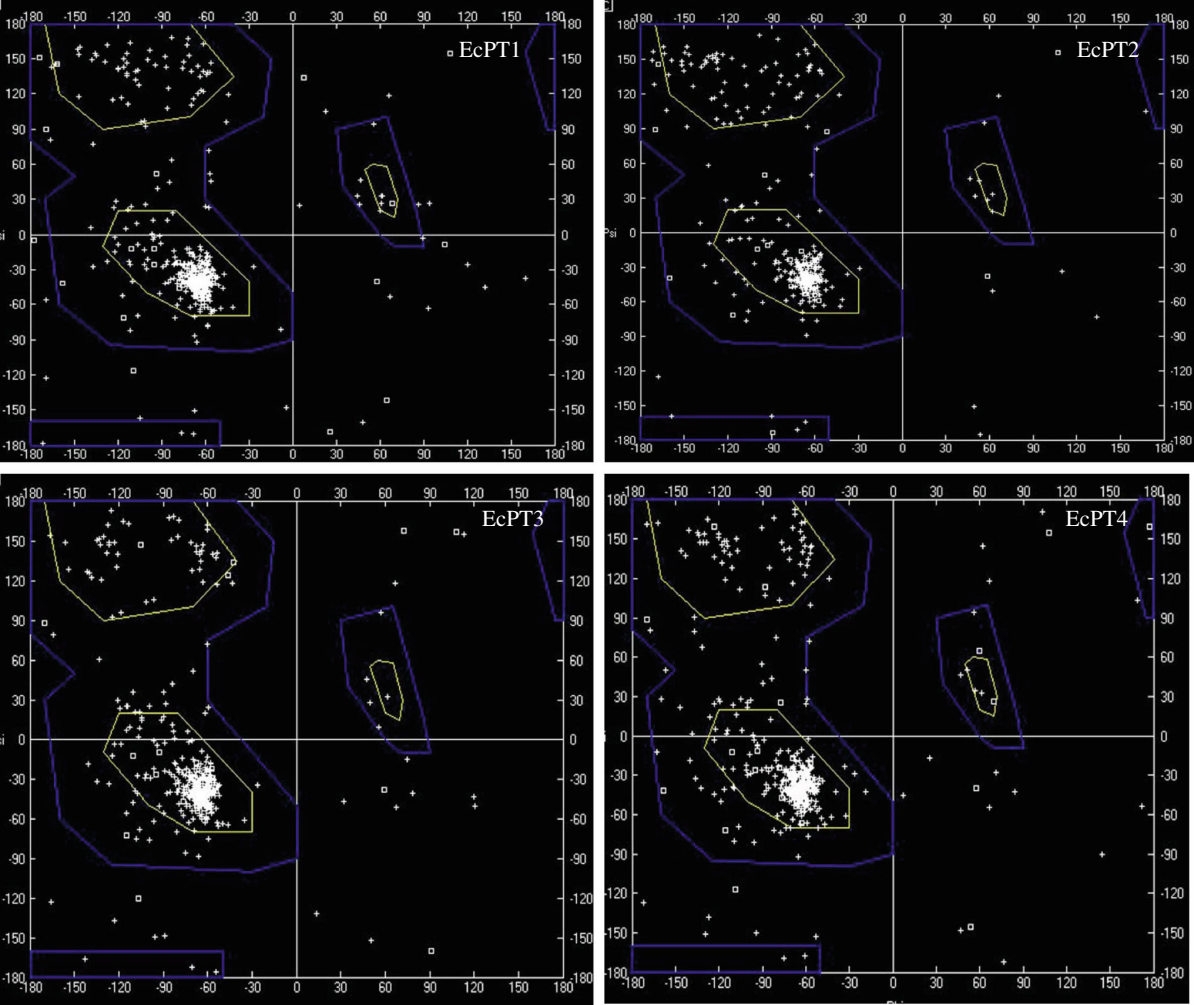
Ramchandran plot of EcPT proteins. The Ramchandran plot of modeled EcPT protein was generated by Swiss PDB viewer. The *x*-*axis* represents φ angle and *y*-*axis* represents Ψ angle. The *plot* shows the presence of favorable alpha-helices and beta sheet (*white*). Majority of amino acids were felled in the region which indicates the stability of the structures of EcPT proteins

The four *EcPT* genes had a significant sequence identity among themselves at the nucleic acid level (from 40.75 to 63.81%) and at the deduced amino acid level (from 43.7 to 69.5%). The estimated molecular masses of *EcPT1, EcPT2, EcPT3 and EcPT4* were 57.09, 44.17, 51.03 and 59.38 kDa, respectively (Table 2). Multiple sequence alignment of EcPT protein shows the presence of several conserved domains. The conserved domains were A-I-V-I-A-G-M-G-F-x-F-T-D-x–Y-D-L-F-S-I, G-R-x–Y-Y, L-C-F–F-R-F-x-L-G-x-G-I-G–G-D-Y-P-L-S-A-T-I-M-S-E-Y-A-N-K, R-G-A-F-I-A-A-V-F-x-M-Q-G, T-Y-Y-W-R-M-x-M-P-E-T-A-R-Y–T-A-L-I/V, and N-x-G-P-N-x-T–T-F-I-x-P-A-E-x-F-P (Fig. 1). The molecular structure of EcPT proteins was modeled using Swiss automatic modeling program (Fig. 2). All the four structures resemble the molecular structure of eukaryotic phosphate transporter protein. The EcPT contains 12 transmembrane helices that contain a central cytosolic tunnel to transfer the phosphate molecule (Fig. 3). Ramchandran plot analysis shows the presence of alpha-helix and beta strand in the favored region (Fig. 4).

**Table 2:** Deduced amino acid and protein information of EcPT proteins

PT proteins	No. of amino acids	Mol. wt. (kDa)	pI
EcPT1	524	57.093	7.80
EcPT2	397	44.171	9.14
EcPT3	470	51.034	9.19
EcPT4	549	59.384	8.31

**Fig. 5:**
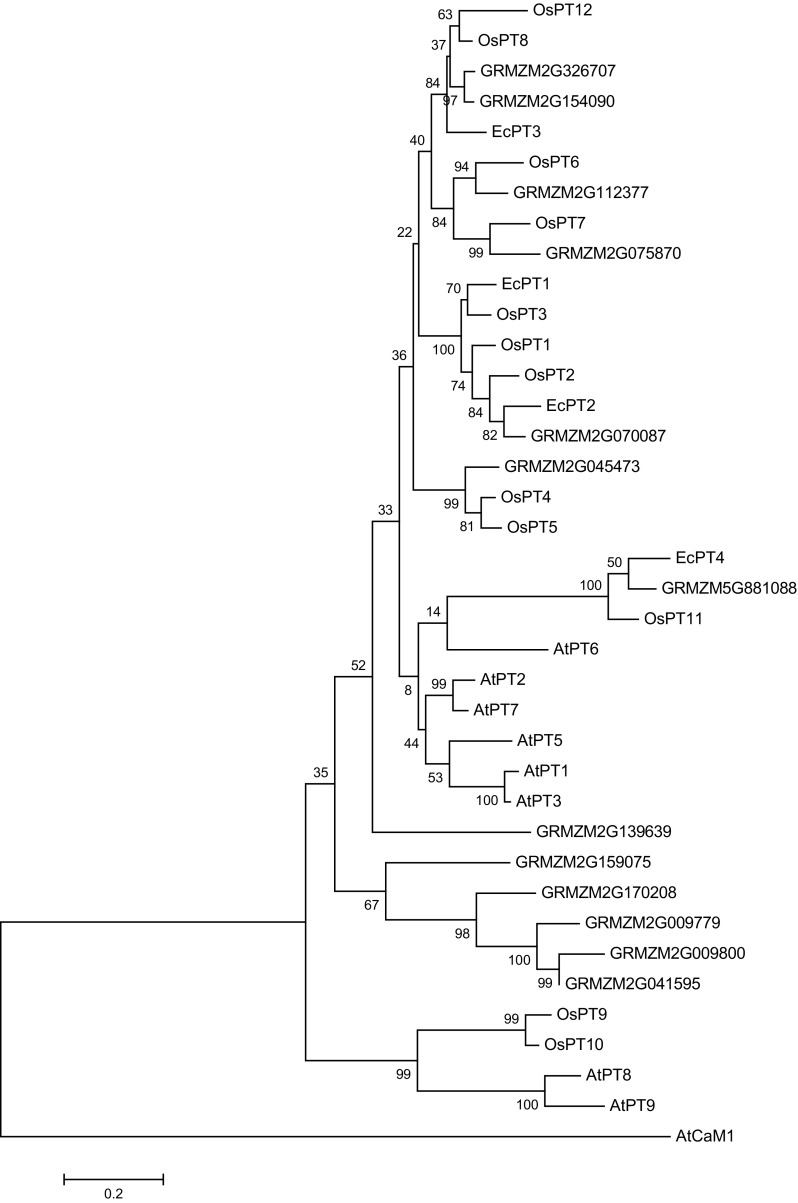
Phylogenetic tree for amino acid sequences of phosphate transporter family members of rice and *EcPT* proteins. The phylogenetic tree was generated by MEGA 4.0 based on a ClustalW2 alignment and the neighbor-joining method for construction of phylogeny (Goujon et al. 2010; Tamura et al. 2007). The branch lengths are proportional to the phylogenetic distances. The sequences of *Oryza sativa* (Paszkowski et al. 2002)*, Zea mays* (Alexandrov et al. 2009; Schnable et al. 2009) and *Arabidopsis thaliana* (Erfle et al. 2000; Mayer et al. 1999) phosphate transporter genes have accession number as *OsPT1* (Q8H6H4), *OsPT2* (Q8GSD9), *OsPT3* (AAN39044), *OsPT4* (Q01MW8), *OsPT5* (AAN39046), *OsPT6* (NP_001062527), *OsPT7* (AAN39048), *OsPT8* (AAN39049) *OsPT9* (AAN39050), *OsPT10* (AAN39051), *OsPT11* (AAN39052), *OsPT12* (AAN39053), *OsPT13* (AAN39054), *GRMZM2G070087* (NM_001196972), *GRMZM2G326707* (NM_001279426), *GRMZM2G154090* (NP_001105816), *GRMZM2G112377* (NP_001105817), *GRMZM2G045473* (NP_001132684), *GRMZM2G075870* (NP_001151202), *GRMZM2G139639* (NP_001149892), *GRMZM5G881088* (NP_001105776), GRMZM2G170208 (NP_001266911), GRMZM2G159075 (AFW57855), GRMZM2G041595 (DAA64043), GRMZM2G009779 (XP_008669651), GRMZM2G009800 (DAA38524), AtPT1 (NP_199149), AtPT2 (NP_181428), AtPT3(NP_199150), AtPT5(NP_180842), AtPT6 (NP_199148), AtPT7 (NP_191030), AtPT8 (NP_173510), AtPT9 (NP_177769). The accession number for AtCaM1 is NP_001154755. The accession numbers of *EcPT1, EcPT2, EcPT3* and *EcPT4* are KJ842583, KJ842584, KJ842585 and KJ842586, respectively

The proteins were found to be hydrophobic in nature and were predicted to be localized to membrane (WoLF PSORT prediction) (Yu et al. 2010). *EcPT1, EcPT2, EcPT3 and EcPT4* were containing 12, 9, 6 and 11 membrane-spanning domains, respectively, as reported by TMHMM (http://www.cbs.dtu.dk/services/TMHMM/) prediction (Fig. 3). The BlastP searches against GenBank data base indicate that *EcPT1*-*4* share 82 to 92% similarity in amino acid with phosphate transporters from *Sorghum bicolor* (Accession number XP_002467158) (Zheng et al. 2011), *Zea mays* (Accession number NP_001183901) (Soderlund et al. 2009), *Sorghum bicolor* (Accession number XP_002464558) (Zheng et al. 2011), and *Zea mays* ZmPT6 (Accession number NP_001105776) (Soderlund et al. 2009). When phylogenetic tree analysis was carried out to compare the EcPT genes with the rice, maize and Arabidopsis phosphate transporter genes (Fig. 5); it was found that *EcPT 1* and 2 was located in group of OsPT- 1, 2, 3, and GRMZM2G070087 while *EcPT3* was found on separate branch of phylogenetic tree along with OsPT- 8,12, GRMZM2G326707, and GRMZM2G154090. *EcPT4* was in the group of OsPT 11 and GRMZM5G881088.

### Expression of *EcPT1*-*4* under the AMF symbiosis

**Fig. 6:**
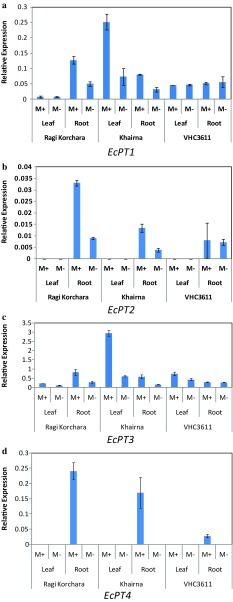
Relative expressions of EcPT1-4 genes in Finger millet leaves and roots of non-mycorrhizal roots and mycorrhizal plants of three varieties (Ragi Korchara Local, Khairna and VHC 3611) after inoculation with *G. intraradices*. **a**–**d** Represents the relative expressions of *EcPT1*-*4*, respectively. Gene expression was analyzed by Real-Time qRT-PCR for three biological replicates of uninoculated plants (M−) and of AM plants (M+) using the specific primers listed in Table 1. The *Ct* values (threshold cycles) of the samples were normalized by the *Ct* values of housekeeping gene *EcTub*. The data for each condition are presented as the mean ± SD and were obtained from three biological and three technical replicates

Results of real-time qRT-PCR with gene-specific primers (Table 1) showed the increased relative transcript abundance of *EcPT1* in roots of Ragi Korchara Local roots and leaves of Khairna variety seedlings when colonized with mycorrhizal fungi. However, the transcript abundance hardly changed in VHC3611, as it was same with and without mycorrhiza (Fig. 6a). The relative transcript abundance levels of *EcPT2* were significantly higher in roots of AM plants of Ragi Korchara Local and Khairna than in non-mycorrhizal plants, and its expression was similar in case of VHC3611 as it was same in roots with and without mycorrhiza. We were unable to detect *EcPT2* transcripts in leaves of all three varieties (Fig. 6b). Transcript accumulation pattern of *EcPT3* was similar to *EcPT1*, where its transcript abundance was six times more in leaves of mycorrhizal seedlings of Khairna as compared to non-mycorrhizal plants. Its expression level did not change in roots and leaves of VHC3611 (Fig. 6c). The expression pattern of *EcPT4* indicated that it was a mycorrhiza-specific phosphate transporter of finger millet and expressed only in mycorrhizal roots of all three varieties (Fig. 6d). The expression study in three varieties under mycorrhizal colonization revealed that the expressions of *EcPT1*-4 showed variable pattern, and their expression was found related to percent colonization of roots by mycorrhiza. Percentage of total colonization by *G*. *intraradices* after 30 days of infection was 95, 60, and 50% in Ragi Korchara Local, Khairna and VHC 3611, respectively. The colonization level of was more in case of Ragi Korchara Local and Khairna as compare to VHC3611, and also the higher expression of PT genes (Fig. 6).

### Regulation of *EcPT1*-*4*in response to phosphorus stress

**Fig. 7:**
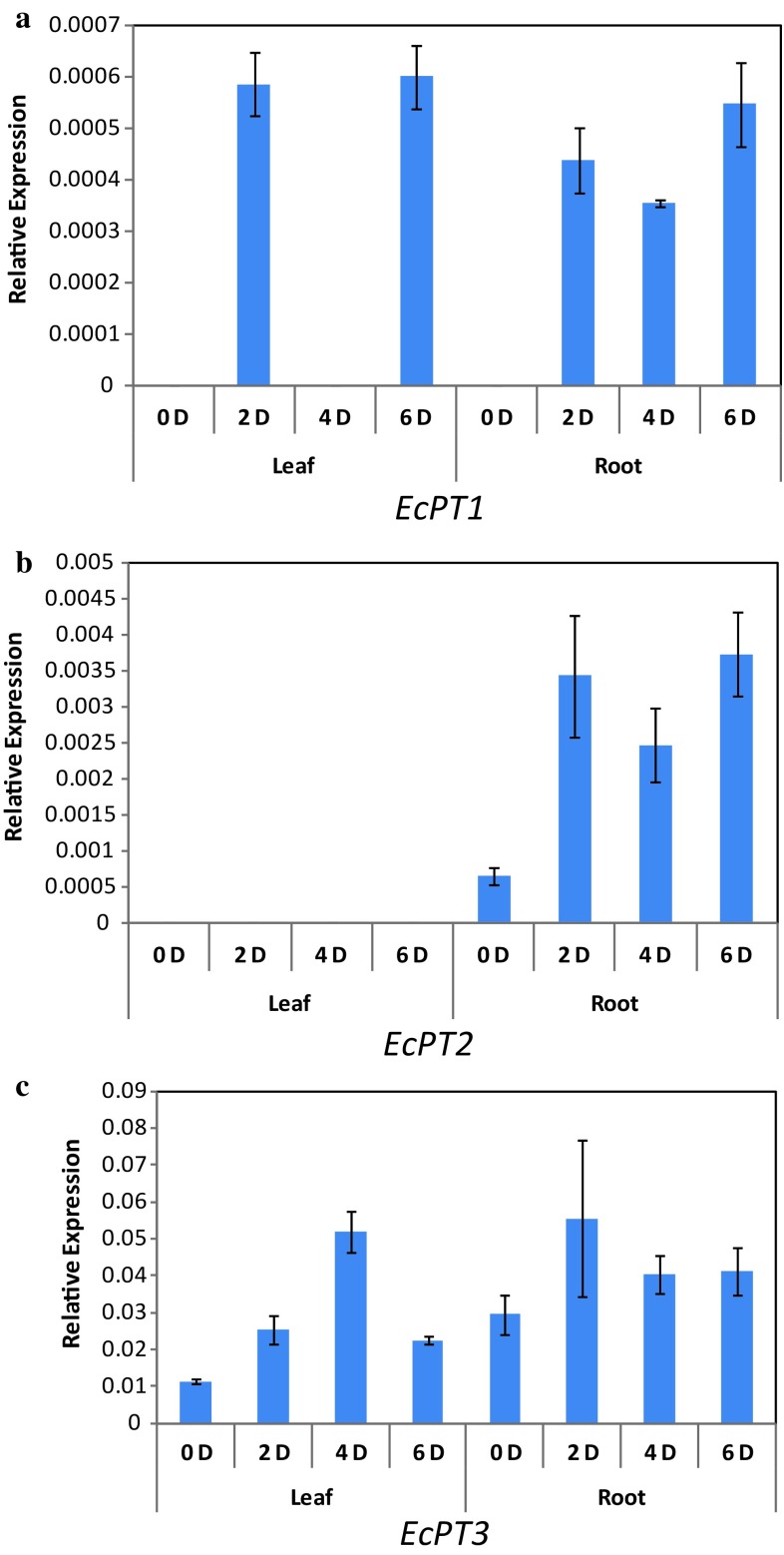
Relative expression of four *EcPT1*-*4* genes in Finger millet leaves and roots under Pi stress. **a**–**c** Represents the relative expressions of *EcPT1*-*3*, respectively. Gene expression was analyzed by Real-Time qRT-PCR for three replicates of plants harvested at 0–6 days of Pi stress using the specific primers listed in Table 1. The expression study was unable to detect the transcript of *EcPT4* gene. Tubulin gene (*EcTub*) was used as internal control

To determine whether the expression of the EcPT genes cloned in this study is related to phosphorus stress, the transcription was analyzed by qRT-PCR in the root and leaves of seedlings grown under the Pi stress. Figure 7 shows the expression level and it indicates that the transcript level of the phosphate transporters in finger millet was highly variable. A comparison of the normalized *EcPT1* transcript levels revealed that transcripts were ~ fivefold higher in roots and leaves when grown at lesser P level for 6 days, compared with the control treatment (Fig. 7a). This shows that the EcPT1 gene was highly active due to Pi starvation. *EcPT2* transcripts were undetectable in leaves of seedlings when grown under normally and also under Pi stress. But its transcripts had shown a significant increase in roots of the seedlings grown under Pi stress (Fig. 7b). *EcPT3* gene was found to be more responsive to phosphate stress and its transcript level was significantly increased in the beginning of treatment in both leaves and roots, but reduced over the period of time of treatment (Fig. 7c). *EcPT4* expression was not detected in Pi stress (Data not shown). The results indicated that there was a significant induction of phosphate transporter transcripts in response to phosphate starvation in seedlings.

## Discussion

Based on the sequence of rice phosphate transporter genes, two complete and two partial phosphate transporter genes were cloned from finger millet. Compared with conserved domain database at NCBI (Marchler-Bauer et al. 2011), it was found that Pht1 family members of finger millet contain the characteristic domains specific to the Major Facilitator Superfamily (MFS) protein family which is a major class of membrane proteins (Abramson et al. 2003). The genes cloned in this study also showed conserved multi-domains of phosphate uptake transporter subfamily of the MFS (Marchler-Bauer et al. 2011), and this indicates that the cloned genes were members of phosphate transporter protein family. The phylogenetic tree analysis showed that *EcPT* genes showed homology with PT genes of rice and maize (Fig. 5), where *EcPT*-*1 and 2* were found in the group of *OsPT*- *1, 2, 3*, and *GRMZM2G070087*, while *EcPT3* was found along with OsPT- 8, 12, GRMZM2G326707, and GRMZM2G154090*. EcPT4* showed closeness to OsPT 11 and GRMZM5G881088, and interestingly *OsPT11* has reported to be mycorrhiza specific (Paszkowski et al. 2002). The same results were found in our study, which confirm that physiological processes involved in AMF- plant symbiosis seem to be conserved during the process of evolution. The presence of conserved domains in EcPT and OsPT protein showed that the proteins are monocot specific and might have evolved from their common ancestors. The molecular structure of EcPT protein resembles the molecular structure of eukaryotic phosphate transporter protein. This signifies that these proteins are evolved for common functionalities of phosphate transportation in plant lineage.

We investigated root colonization, and transcription of genes *EcPT1*-*4* after inoculation with *G. intraradices* and Pi stress in finger millet. From the results, it was clear that the rate of colonization by mycorrhiza was variety specific and some varieties of finger millet are more responsive to mycorrhiza infection (Unpublished data). Also, the increase in root colonization was related with the increased expression of phosphate transporter genes cloned in this study. The previous studies have supported our findings that AM can increase the phosphate transport in plants by increasing the activity of host phosphate transporter genes (Nagy et al. 2005; Tan et al. 2012).

During the expression study, a change in the expressions of *EcPT1*-*4* was detected in three different varieties of finger millet with AMF infection. *EcPT4* did not express in leaves and roots of non-mycorrhizal seedlings of all three varieties, but only expressed in roots infected with AM. Under the Pi stress also we were unable to detect its transcript in Ragi Korchara Local. Previous studies have shown that in plants some of the PT genes are induced by AM colonization in roots (Karandashov et al. 2004; Nagy et al. 2005; Paszkowski et al. 2002; Siciliano et al. 2007; Tan et al. 2012; Wegmuller et al. 2008). The results of our transcript abundance study indicated that *EcPT4* was mycorrhiza-specific finger millet phosphate transporter gene and expressed with inoculation of with *G. intraradices*.

The finger millet seedlings grown in the liquid nutrient media with lower P concentrations have shown a different expression pattern of phosphate transporter genes. The variable rate of transcript abundance may be due to various factors like promoter controlling the responsiveness to Pi stress (Liu et al. 1998a). Results of the expression in leaves in all three varieties indicated that various genetic and spatial factors influence phosphate transporter activity during the plant P uptake (Inoue et al. 2014). These results suggested that the cloned genes might be involved in diverse processes along with the direct uptake pathway of P by membrane-spanning phosphate transporters (Bayle et al. 2011; Inoue et al. 2014).

## Conclusion

In the present study, cloning of four phosphate transporter family genes *EcPT1*-*4* from finger millet was achieved, and expression study under the mycorrhiza colonization and Pi stress was conducted to characterize them. The results showed that out of four genes cloned in this study, *EcPT4* is the mycorrhiza-specific PT gene and its expression level is correlated with the percentage of root colonization by AM. Additionally, we found that the pattern of the expression of these genes under Pi stress was different and needs to be further investigated to check out the causes for this differential expression during phosphorus uptake and plant growth. Our results support the conservation of functional role of some of the family genes during the period of evolution, which was the case for *EcPT4*.

## Abbreviations

Pht: Phosphate transporter
AM: Arbuscular mycorrhiza
RT: Reverse transcription
qRT-PCR: Quantitative real-time PCR
